# Conformal mirror descent with logarithmic divergences

**DOI:** 10.1007/s41884-022-00089-3

**Published:** 2022-12-14

**Authors:** Amanjit Singh Kainth, Ting-Kam Leonard Wong, Frank Rudzicz

**Affiliations:** 1 Department of Computer Science, University of Toronto, Toronto, Canada; 2 Department of Statistical Sciences, University of Toronto, Toronto, Canada; 3 Vector Institute for Artificial Intelligence, Toronto, Canada

**Keywords:** Mirror descent, Gradient flow, Logarithmic divergence, Conformal Hessian metric, $\lambda$-duality, $\lambda$-exponential

## Abstract

The logarithmic divergence is an extension of the Bregman divergence motivated by optimal transport and a generalized convex duality, and satisfies many remarkable properties. Using the geometry induced by the logarithmic divergence, we introduce a generalization of continuous time mirror descent that we term the conformal mirror descent. We derive its dynamics under a generalized mirror map, and show that it is a time change of a corresponding Hessian gradient flow. We also prove convergence results in continuous time. We apply the conformal mirror descent to online estimation of a generalized exponential family, and construct a family of gradient flows on the unit simplex via the Dirichlet optimal transport problem.

## Introduction

Information geometry provides not only powerful tools for studying spaces of probability distributions, but also a wide range of geometric structures that are useful for various challenges in data science [1–3]. The Bregman divergence [4] plays a key role in the theory and application of information geometry. It is the canonical divergence of the dually flat geometry [5] which arises naturally in exponential families [6], and can serve as a loss function in statistical estimation and optimal control [7]. The Bregman divergence is especially tractable in applied settings, as it is closely connected to convex duality and satisfies a generalized Pythagorean theorem that greatly simplifies the analysis of Bregman projections. Among the many applications of Bregman divergences, we mention clustering [6], exponential family principal component analysis [8] as well as boosting and logistic regression [9, 10].

We present in this paper a generalization of *mirror descent* [11, 12], which is a fundamental first-order iterative optimization algorithm. Mirror descent is a gradient descent algorithm where a Bregman divergence serves as a proximal function. A suitable convex generating function may be chosen to exploit the geometry of the problem. The update step (6) of mirror descent involves a change of coordinates using the so-called *mirror map* which corresponds to the information-geometric dual parameter. In the continuous time limit, mirror descent can be represented as a Riemannian gradient flow with respect to the Hessian metric induced by the given Bregman divergence [13, 14]. The basic ideas are reviewed in Sects. 2.1 and 2.3.

Our generalization, termed the *conformal mirror descent*, is based on the theory of *logarithmic divergences* [15–19]. In many senses, the logarithmic divergence may be regarded as a canonical deformation of the Bregman divergence. Just as the Bregman divergence captures the dually flat geometry, the logarithmic divergence is a canonical divergence for a dually projectively flat statistical manifold with *constant* nonzero sectional curvature, and also satisfies a generalized Pythagorean theorem [17]. Moreover, the logarithmic divergence leads, under divisive normalization, to a deformed exponential family, which is closely related to the *q*-exponential family in statistical physics [20], while recovering natural analogues of information-geometric properties of the exponential family in the deformed case [17, 19]. For example, the Kullback-Leibler (KL) divergence (which is the Bregman divergence of the cumulant generating function) becomes the Rényi divergence, and the dual variable can be interpreted as an escort expectation. Another appealing property is that the logarithmic divergence is associated with a generalized convex duality motivated by optimal transport [21, 22]. Following [19], we call it the $\lambda$-*duality*, where $\lambda \ne 0$ is the curvature parameter. It was recently shown [23] that the dualistic geometry in information geometry can be naturally embedded in the pseudo-Riemannian geometry of optimal transport [24] using the framework of *c*-*divergence*, under which divergences are induced by optimal transport maps. Bregman and logarithmic divergences are special cases corresponding to specific cost functions [16, 17]. In Sect. 2.4, we review properties of $\lambda$-duality and logarithmic divergences that are needed in this paper. Further results about $\lambda$-duality and its relation with convex duality can be found in [25].

In Sect. 3, we formulate the conformal mirror descent in continuous time as a Riemannian gradient flow, where the underlying metric is induced by a logarithmic divergence. We call it the *conformal mirror descent* because the metric can be shown to be a conformal transformation of a Hessian metric. This implies that the conformal mirror descent is, in continuous time, a time-changed mirror descent with respect to an explicitly determined convex generator. We also derive explicit dynamics of the gradient flow under the $\lambda$-mirror map corresponding to the logarithmic divergence and prove related convergence results. The $\lambda$-duality suggests many new generating functions that are potentially useful in various applications.

We give two applications to demonstrate the utility of our conformal mirror descent. In Sect. 4, we consider online estimation of the $\lambda$-exponential family introduced in [19], and derive an elegant online natural gradient update which generalizes the one for the exponential family [14]. *Dirichlet optimal transport* on the unit simplex [15, 16, 26] is one of the original motivations of the theory of logarithmic divergences (and corresponds to the case $\lambda = -1$). Expressing the $(-1)$-mirror map in terms of the Dirichlet optimal transport map, we derive in Sect. 5 an interesting family of gradient flows on the unit simplex.

Finally, in Sect. 6 we discuss our contributions in the context of related literature, and propose several directions for future research.

*Notation*: We use superscripts to denote components of vectors, e.g., $\theta = (\theta ^1, \ldots , \theta ^d)$. In computations, we regard $\theta$ as a column vector and write $$\theta = \begin{bmatrix} \theta ^1&\cdots&\theta ^d\end{bmatrix}^{\top }$$, where $\top$ denotes transposition. The Euclidean gradient ${\textsf{D}} f(\theta ) = {\textsf{D}}_{\theta } f(\theta )$ of a real-valued function *f* is also regarded as a column vector. The Euclidean Hessian is denoted by ${\textsf{D}}^2 f(\theta )$. Due to the difficulty of unifying notations in different settings, in this paper we do *not* adopt the Einstein summation convention.

## From convex duality to $\lambda$-duality

### Convex duality and Bregman divergence

We begin by reviewing convex duality and Bregman divergence, which are at the core of classical information geometry [1, 2] (also see [27] for a recent overview). Let $$\phi $$ be a lower semicontinuous convex function on $$\mathbbm {R}^d$$. Its convex conjugate is defined by $$\phi ^*(y) = \sup _{x \in \mathbbm {R}^d} \left\{ {\left\langle x, y\right\rangle } - \phi (x) \right\} $$, where $${\left\langle \cdot , \cdot \right\rangle }$$ denotes the Euclidean inner product. Then $$\phi ^*$$ is also lower semi-continuous and convex, and we have $$\phi ^{**} = (\phi ^*)^* = \phi $$. For any $$x, y \in \mathbbm {R}^d$$ we have

1 $$\begin{aligned} \phi (x) + \phi ^*(y) - {\left\langle x, y\right\rangle } \ge 0, \end{aligned}$$

and equality holds if and only if *y* is a subgradient of $\phi$ at *x*.

Let $\Theta \subset \mathbbm {R}^d$ be an open convex set and let $\phi : \Theta \rightarrow \mathbbm {R}$ be a smooth convex function whose Hessian ${\textsf{D}}^2 \phi (\theta )$ is everywhere positive definite. We call such a $\phi$ a *Bregman generator*. The *Bregman divergence* of $\phi$, regarded as a generalized distance, is defined for $\theta , \theta ' \in \Theta$ by

2 $$\begin{aligned} \textbf{B}_{\phi }[\theta : \theta '] = \left( \phi (\theta ) - \phi (\theta ')\right) - {\left\langle {\textsf{D}} \phi (\theta '), \theta - \theta '\right\rangle }. \end{aligned}$$

Under the stated conditions, ${\textsf{D}} \phi$ is a diffeomorphism from $\Theta$ onto its range. We call $\theta$ the *primal variable* and $\zeta = {\textsf{D}} \phi (\theta )$ the *dual variable*.^1^ The inverse transformation is given by $\theta = {\textsf{D}} \phi ^*(\zeta )$. The Bregman divergence (2) can then be expressed in *self-dual form* by

3 $$\begin{aligned} \begin{aligned} \textbf{B}_{\phi }[\theta : \theta '] = \textbf{B}_{\phi ^*}[\zeta ' : \zeta ] = \phi (\theta ) + \phi ^*(\zeta ') - {\left\langle \theta , \zeta '\right\rangle }, \end{aligned} \end{aligned}$$

which is closely related to the *Fenchel-Young inequality* (1).

### *c*-duality

Conjugation, which characterizes convex duality, is defined in terms of the *linear* pairing function $c(x, y) = -{\left\langle x, y\right\rangle }$. It turns out that much of the above can be generalized. For a general *c*, called a cost function in the context of optimal transport [21, 22], we can define the *c*-conjugate of a function $\varphi (x)$ by $\varphi ^{(c)}(y) = \sup _{x} \{ -c(x, y) - \varphi (x) \}$. A function $\varphi (x)$ is said to be *c*-convex if it is the *c*-conjugate of some function $\psi (y)$, i.e., $\varphi = \psi ^{(c)}$ (*c*-convexity of $\psi (y)$ is defined similarly). For a *c*-convex $\varphi (x)$ we have the following analogue of the Fenchel-Young inequality (1):

4 $$\begin{aligned} \varphi (x) + \varphi ^{(c)}(y) + c(x, y) \ge 0. \end{aligned}$$

If equality holds, we call *y* a *c*-subgradient of $\varphi$ at *x*. If this *y* is unique, we call it the *c*-gradient and write $y = {\textsf{D}}^{(c)} \varphi (x)$. Under suitable conditions, a Monge-Kantorovich optimal transport problem can be solved by an optimal transport map, which can be expressed as the *c*-gradient of some *c*-convex potential $\varphi$. Analogous to (3), the inequality (4) can be used to define a *c-divergence* on the graph of the optimal transport map [23]. The $\lambda$-duality [19] is the generalized convex duality based on the *logarithmic cost*

5 $$\begin{aligned} c_{\lambda }(x, y) = \frac{-1}{\lambda } \log (1 + \lambda {\left\langle x, y\right\rangle }), \end{aligned}$$

where $\lambda$ is a given nonzero constant.^2^ Since $\lim _{\lambda \rightarrow 0} c_{\lambda }(x, y) = - {\left\langle x, y\right\rangle }$, we recover the usual convex duality when $\lambda \rightarrow 0$. Relevant properties of the $\lambda$-duality will be reviewed in Sect. 2.4.

### Mirror descent

Consider the minimization problem $\min _{\theta \in \Theta } f(\theta )$ where $f: \Theta \rightarrow \mathbbm {R}$ is assumed to be differentiable. Let $\phi : \Theta \rightarrow \mathbbm {R}$ be a Bregman generator as in Sect. 2.1. It induces the *mirror map* $\zeta = {\textsf{D}}_{\theta } \phi (\theta )$. For clarity, we use ${\textsf{D}}_{\theta }$ to indicate that the gradient is taken with respect to $\theta$. The mirror descent algorithm minimizes *f* by iterating the update

6 $$\begin{aligned} \zeta _{k+1} = \zeta _k - \delta {\textsf{D}}_{\theta } f(\theta _k), \end{aligned}$$

where $\delta = \delta _k > 0$ is the learning rate which may depend on *k*. We obtain $\theta _{k+1}$ by applying the inverse mirror map, i.e., $\theta _{k+1} = {\textsf{D}}_{\zeta } \phi ^*(\zeta _{k+1})$. To implement the algorithm, we usually require that both ${\textsf{D}} \phi$ and ${\textsf{D}} \phi ^*$ are available in closed form. Letting $\phi (\theta ) = \frac{1}{2} \vert \theta \vert ^2 = \frac{1}{2} {\left\langle \theta , \theta \right\rangle }$ recovers Euclidean gradient descent since in this case, $\zeta = {\textsf{D}}_{\theta } \frac{1}{2}\vert \theta \vert ^2 = \theta$. In general, (6) requires an extra projection step when the right hand side is outside $\Theta$. The (unconstrained) update (6) is equivalent to the update of a *Bregman proximal method*, namely

7 $$\begin{aligned} \theta _{k+1} = {{\,\mathrm{\textsf{argmin}}\,}}_{\theta \in \Theta } \left\{ f(\theta _k) + {\left\langle {\textsf{D}}_{\theta } f(\theta _k), \theta - \theta _k\right\rangle } + \frac{1}{\delta } \textbf{B}_{\phi }[\theta : \theta _k] \right\} . \end{aligned}$$

It is easy to verify that the first order condition of (7) can be expressed as (6). Geometrically, $\theta _{k+1}$ minimizes a linear approximation of *f* over a Bregman ball based at $\theta _k$.

Further insights can be obtained by studying the continuous time limit as in [14, 28]. The Bregman divergence admits the quadratic approximation

8 $$\begin{aligned} \textbf{B}_{\phi }[ \theta + \Delta \theta : \theta ] = \frac{1}{2} (\Delta \theta )^{\top } G_0(\theta ) (\Delta \theta ) + O(\vert \Delta \theta \vert ^3), \end{aligned}$$

where $G_0(\theta ) = {\textsf{D}}_{\theta }^2 \phi (\theta )$ is a *Hessian* Riemannian metric (expressed under the primal $\theta$-coordinates) and induces the *Riemannian gradient* $\textrm{grad}_{G_0} f = G_0^{-1} {\textsf{D}}_{\theta } f$ (in coordinates). See [29] for an in-depth geometric study of Hessian manifolds. Letting $\delta \rightarrow 0$ in (6) or (7) and scaling time appropriately, one obtains a *Hessian Riemannian gradient flow* [13]:

9 $$\begin{aligned} \begin{aligned} {\frac{\text {d}}{\text {d}{t}}} \theta _t = -\textrm{grad}_{G_0} f(\theta _t), \quad \text {or equivalently} \quad {\frac{\text {d}}{\text {d}{t}}} \zeta _t = -{\textsf{D}}_{\theta } f(\theta _t). \end{aligned} \end{aligned}$$

Naturally, one may consider other metrics to obtain generalizations of mirror descent (see [28] for a discussion). In this paper, we use the Riemannian metric induced by the logarithmic divergence, which is particularly tractable.

### $\lambda$-duality and logarithmic divergence

In this subsection we introduce the $\lambda$-duality which utilizes the logarithmic cost function $c_{\lambda }$ defined by (5). For more details we refer the reader to [17, 19] on which this work is based. In general, *c*-convex functions and *c*-gradients are not analytically tractable. Remarkably, for the logarithmic cost function $c_{\lambda }$, it is possible to relate $c_{\lambda }$-convexity with usual convexity and express the $c_{\lambda }$-gradient in terms of the usual gradient. The following definition summarizes the generalized convexity notion and the required regularity conditions needed for our applications. Throughout, we let $\lambda \ne 0$ be a fixed constant.

#### Definition 1

(*Regular* $c_{\lambda }$-*convex function and* $c_{\lambda }$-*gradient*) Let $\Theta \subset \mathbbm {R}^d$ be an open convex set. A smooth function $\varphi : \Theta \rightarrow \mathbbm {R}$ is said to be regular $c_{\lambda }$-convex if $\Phi _{\lambda } = \frac{1}{\lambda } (e^{\lambda \varphi } - 1)$ is a Bregman generator and $1 - \lambda {\left\langle {\textsf{D}}_{\theta } \varphi (\theta ), \theta \right\rangle } > 0$ on $\Theta$. Given such a function $\varphi$, we define its $c_{\lambda }$-gradient by

10 $$\begin{aligned} {\textsf{D}}^{(c_{\lambda })}_{\theta } \varphi (\theta ) = \frac{1}{1 - \lambda {\left\langle {\textsf{D}}_{\theta } \varphi (\theta ), \theta \right\rangle }} {\textsf{D}}_{\theta } \varphi (\theta ). \end{aligned}$$

By (11) below, the right hand side of (10) is indeed the $c_{\lambda }$-gradient of $\varphi$ as a $c_{\lambda }$-convex function. We also call ${\textsf{D}}^{(c_{\lambda })}_{\theta } \varphi$ the $\lambda$-*mirror map*. Under the stated conditions, it can be shown that ${\textsf{D}}^{(c_{\lambda })}_{\theta } \varphi$ is a diffeomorphism from $\Theta$ onto its range *H*;^3^ we call $\eta = {\textsf{D}}^{(c_{\lambda })} \varphi (\theta )$ the dual variable in this context. In a nutshell, instead of convex functions, we use functions $\varphi$ such that $\Phi _{\lambda } = \frac{1}{\lambda } (e^{\lambda \varphi } - 1)$ are convex, and replace the usual gradient by the $\lambda$-mirror map defined by (10). Some examples of regular $c_{\lambda }$-convex functions are given in Table .

**Table 1:** Examples of regular $c_{\lambda }$-convex functions on the real line and their corresponding $\lambda$-mirror maps

$\lambda$ range	$\Theta$	$\varphi (\theta )$	$\eta = {\textsf{D}}^{(c_{\lambda })} \varphi (\theta )$
$(-2, \infty )$	$(0, \infty )$	$-\frac{1}{2} \log \theta$	$\frac{-1}{2 + \lambda } \frac{1}{\theta }$
$(0, \infty )$	$\left( -\infty , \frac{1}{\lambda }\right)$	$\theta$	$\frac{1}{1 - \lambda \theta }$
$\mathbbm {R} {\setminus } \{0\}$	$\left( \frac{-1}{\sqrt{\vert \lambda \vert }}, \frac{1}{\sqrt{\vert \lambda \vert }}\right)$	$\frac{1}{2}\theta ^2$	$\frac{\theta }{1 - \lambda \theta ^2}$

Henceforth we let $\varphi$ be a regular $c_{\lambda }$-convex function on a given convex domain $\Theta$. Let $\psi$ be the $c_{\lambda }$-*conjugate* defined by

$$\begin{aligned} \psi (\eta ) = \sup _{\theta \in \Theta } \left\{ -c_{\lambda }(\theta , \eta ) - \varphi (\theta ) \right\} . \end{aligned}$$

Then, for $\theta \in \Theta$ we have

$$\begin{aligned} \varphi (\theta ) = \sup _{\eta \in H} \left\{ -c_{\lambda }(\theta , \eta ) - \psi (\eta ) \right\} . \end{aligned}$$

Hence, $\varphi$ is a $c_{\lambda }$-convex function in the sense of Sect. 2.2. We have $1 + \lambda {\left\langle \theta , \eta '\right\rangle } > 0$ for any $(\theta , \eta ') \in \Theta \times H$, and for $\eta = {\textsf{D}}^{(c_{\lambda })} \varphi (\theta )$ we have

11 $$\begin{aligned} \varphi (\theta ) + \psi (\eta ) + c_{\lambda }(\theta , \eta ) = 0. \end{aligned}$$

Thus $\varphi$ and $\psi$ satisfy a generalized Legendre-like duality with respect to the cost function $c_{\lambda }$. The inverse $\lambda$-mirror map is given by $\theta = {\textsf{D}}_{\eta }^{(c_{\lambda })} \psi (\eta )$.

We use $\varphi$ to define a $\lambda$-*logarithmic divergence* which is different from the Bregman divergence. For completeness, we explain how it is constructed. Recall that $\Phi _{\lambda } = \frac{1}{\lambda } (e^{\lambda \varphi } - 1)$ is (strictly) convex on $\Theta$. For $\theta , \theta ' \in \Theta$, we have

$$\begin{aligned} \Phi (\theta ') + {\left\langle {\textsf{D}} \Phi (\theta '), \theta - \theta '\right\rangle } \le \Phi (\theta ). \end{aligned}$$

Expressing this inequality in terms of $\varphi$, we have, using the chain rule,

12 $$\begin{aligned} \begin{aligned}&\quad \frac{1}{\lambda } e^{\lambda \varphi (\theta ')} + e^{\lambda \varphi (\theta ')} \langle \textsf {D}\varphi (\theta ') , \theta - \theta ' \rangle \le \frac{1}{\lambda } e^{\lambda \varphi (\theta )}\\ {}&\Rightarrow \frac{1}{\lambda }(1 + \lambda \langle {\textsf {D}} \varphi (\theta ') , \theta - \theta ' \rangle ) \le \frac{1}{\lambda } e^{\lambda (\varphi (\theta ) - \varphi (\theta '))}. \end{aligned} \end{aligned}$$

Now there are two cases depending on the sign of $\lambda$, but the resulting expression is the same. Here, we consider the case $\lambda < 0$ and the other case is similar. From (12), we have

13 $$\begin{aligned} \begin{aligned}&\quad 1 + \lambda \langle {\textsf {D}} \varphi (\theta ') , \theta - \theta ' \rangle \ge e^{\lambda (\varphi (\theta ) - \varphi (\theta '))} \\ {}&\Rightarrow \varphi (\theta ') + \frac{1}{\lambda } \log (1 + \langle \lambda {\textsf {D}} \varphi (\theta ') , \theta - \theta ' \rangle ) \le \varphi (\theta ). \end{aligned} \end{aligned}$$

Taking the difference yields the $\lambda$-logarithmic divergence. When $\varphi$ is convex, letting $\lambda \rightarrow 0$ in (13) recovers the Bregman divergence (see Figure ).

**Fig. 1:**
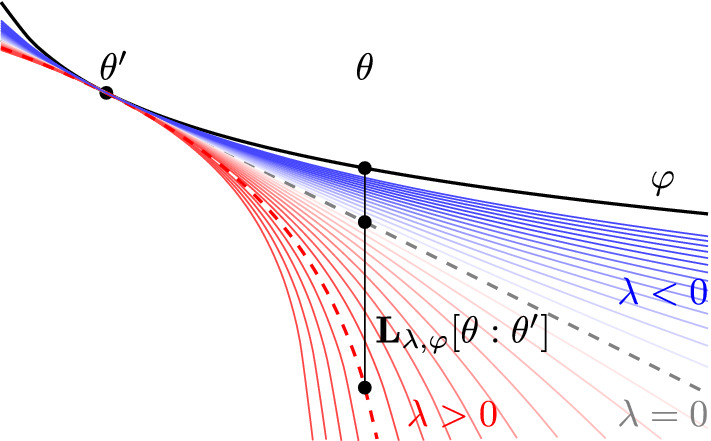
The $\lambda$-logarithmic divergence (14) is the error term of a logarithmic first order approximation; see (13). We visualize it for $\varphi (\theta ) = \frac{-1}{2} \log \theta$ which is regular $c_{\lambda }$-convex on $(0, \infty )$ for $\lambda > -2$. The red dashed curve shows the case $\lambda = 1$

#### Definition 2

($\lambda$*-logarithmic divergence*) Let $\varphi$ be a regular $c_{\lambda }$-convex function. We define its $\lambda$-logarithmic divergence for $\theta , \theta ' \in \Theta$ by

14 $$\begin{aligned} \textbf{L}_{\lambda , \varphi }[\theta : \theta '] = \varphi (\theta ) - \varphi (\theta ') - \frac{1}{\lambda } \log \left( 1 + \lambda {\left\langle {\textsf{D}} \varphi (\theta '), \theta - \theta '\right\rangle }\right) . \end{aligned}$$

Analogous to (3), it also admits a self-dual representation:

15 $$\begin{aligned} \textbf{L}_{\lambda , \varphi }[\theta : \theta '] = \textbf{L}_{\lambda , \psi }[\eta ' : \eta ] = \varphi (\theta ) + \psi (\eta ') - \frac{1}{\lambda } \log \left( 1 + \lambda {\left\langle \theta , \eta '\right\rangle }\right) \ge 0. \end{aligned}$$

This identity verifies that $\textbf{L}_{\lambda , \varphi }$ is the *c*-divergence of the cost $c_{\lambda }$ (see [17, 23]). An important application of the logarithmic divergence is to some generalized exponential families, where an appropriately defined potential function $\varphi$ leads to the Rényi divergence. In Sect. 4, we exploit this property in online parameter estimation.

Similar to (8), we may Taylor expand $\textbf{L}_{\lambda , \varphi }[\theta + \Delta \theta : \theta ]$ to get

$$\begin{aligned} \textbf{L}_{\lambda , \varphi }[ \theta + \Delta \theta : \theta ] = \frac{1}{2} (\Delta \theta )^{\top } G_{\lambda } (\theta ) (\Delta \theta ) + O(\vert \Delta \theta \vert ^3), \end{aligned}$$

where $G_{\lambda }(\theta )$ is the matrix given by

16 $$\begin{aligned} G_{\lambda }(\theta ) = {\textsf{D}}^2_{\theta } \varphi (\theta ) + \lambda ({\textsf{D}}_{\theta } \varphi (\theta ))({\textsf{D}}_{\theta } \varphi (\theta ))^{\top } = e^{-\lambda \varphi (\theta )} {\textsf{D}}_{\theta }^2 \Phi _{\lambda }(\theta ). \end{aligned}$$

Note that the last equality follows from the identity $\Phi _{\lambda } = \frac{1}{\lambda } (e^{\lambda \varphi } - 1)$ and the chain rule. From Definition 1, $G_{\lambda }(\theta )$ is positive definite.

Regard $\theta \in \Theta$ and $\eta = {\textsf{D}}^{(c_{\lambda })} \varphi (\theta ) \in H$ as *global* coordinate systems of a manifold *M*.^4^ On *M*, we define a divergence $\textbf{D}(\cdot \vert \vert \cdot )$ on *M* by

17 $$\begin{aligned} \textbf{D}(P\vert \vert Q) = \varphi (\theta _P) + \psi (\eta _Q) + c_{\lambda }(\theta _P, \eta _Q), \end{aligned}$$

where $\theta _P$ and $\eta _P$ are respectively the primal and dual coordinates of *P* (similar for *Q*). The induced dualistic geometry $(g, \nabla , \nabla ^*)$ (constructed using Eguchi’s theory, see e.g., [30]) has the following remarkable properties [16, 17, 19]:

- The Riemannian metric *g* is given in primal coordinates by
  18 $$\begin{aligned} g\left( \left. \frac{\partial }{\partial \theta ^i}\right| _P, \left. \frac{\partial }{\partial \theta ^j} \right| _P \right) = (G_{\lambda }(\theta _P))_{ij}. \end{aligned}$$
  The first representation in (16) states that $G_{\lambda }$ is a rank-one correction of the Hessian ${\textsf{D}}^2_{\theta } \varphi$. The second representation states that $G_{\lambda }$ is a *conformal transformation* of the Hessian metric ${\tilde{G}}_0 = {\textsf{D}}_{\theta }^2 \Phi _ {\lambda }$. That is, *g* is a *conformal Hessian metric* (when expressed in primal coordinates). Both expressions are useful in our conformal mirror descent. Analogous expressions hold under the dual coordinate system.
- The primal and dual connections $(\nabla , \nabla ^*)$ are *dually projectively flat*. In particular, any primal (resp. dual) geodesic is a time-reparameterized straight line under the primal (resp. dual) coordinate system.
- When $d \ge 2$, the sectional curvatures of $\nabla$ and $\nabla ^*$ with respect to *g* are everywhere *constant* and equal to $\lambda$.
- The *generalized Pythagorean theorem* extends to $\textbf{D}$.
- Given a dualistic structure which is dually projectively flat with constant (nonzero) sectional curvature, one can define (locally) a $\lambda$-logarithmic divergence which induces the given structure. Thus, the $\lambda$-logarithmic divergence can be regarded as a *canonical divergence*.

Letting $\lambda \rightarrow 0$ recovers well-known properties of the dually flat geometry induced by a Bregman divergence.

#### Remark 1

The last expression in (16) may be realized via the identity

19 $$\begin{aligned} \textbf{L}_{\lambda , \varphi }[\theta : \theta '] = \frac{1}{-\lambda } \log \left( 1 + (-\lambda ) e^{-\lambda \varphi (\theta )} \textbf{B}_{\Phi _{\lambda }}[\theta : \theta '] \right) , \end{aligned}$$

which can be verified by a direct computation. It states that the $\lambda$-logarithmic divergence is a monotone transformation of a *left conformal Bregman divergence* [31]. See [32] for more discussion in this direction.

## Conformal mirror descent

In this section, we present our first main contribution: a generalization of continuous time mirror descent as the Riemannian gradient flow with respect to the metric induced by a $\lambda$-logarithmic divergence. In Sect. 3.1, we define the flow and interpret it in two ways: (i) a mirror-like descent under the $\lambda$-mirror map (10), and (ii) a time change of a Hessian gradient flow. It reduces to the continuous time mirror descent (9) in the limit $\lambda \rightarrow 0$. Convergence results are stated and proved in Sect. 3.2.

### The flow and two representations

As described in Sect. 2.3, the usual (Bregman) mirror descent (6) can be understood as (i) a Bregman proximal method (7); or (ii) a (discretization of) the Hessian gradient flow (9). This suggests two ways to generalize the method. Formally, we may replace the Bregman divergence in (7) by a $\lambda$-logarithmic divergence. This leads to the proximal method

20 $$\begin{aligned} \theta _{k+1} = {{\,\mathrm{\textsf{argmin}}\,}}_{\theta \in \Theta } \left\{ f(\theta _k) + {\left\langle {\textsf{D}}_{\theta } f(\theta _k), \theta - \theta _k\right\rangle } + \frac{1}{\delta } \textbf{L}_{\lambda , \varphi }[\theta : \theta _k] \right\} . \end{aligned}$$

Unfortunately, because of the logarithm, the first order condition of (20) cannot be solved explicitly to yield a simple update as in mirror descent (see (6)). We study instead the continuous time Riemannian gradient flow with respect to the metric *g* given by (18), and it turns out that this is much more tractable. We fix $\lambda \ne 0$ and let a regular $c_{\lambda }$-convex generator $\varphi$ be given on the convex domain $\Theta$.

#### Definition 3

(*Conformal mirror descent in continuous time*) Let $f: \Theta \rightarrow \mathbbm {R}$ be a differentiable function to be minimized. Given an initial value $\theta _0 \in \Theta$, the continuous time conformal mirror descent is the Riemannian gradient flow given in primal coordinates by

21 $$\begin{aligned} {\frac{\text {d}}{\text {d}{t}}} \theta _t = -\textrm{grad}_{G_{\lambda }} f(\theta _t), \end{aligned}$$

where $\textrm{grad}_{G_{\lambda }} f = G_{\lambda }^{-1} {\textsf{D}}_{\theta } f$ is the Riemannian gradient expressed in primal coordinates and $G_{\lambda }$ is given by (16).

While any Riemannian metric can be used to define a gradient flow, implementation of the flow in coordinates generally requires computation of the Riemannian gradient $G^{-1}(\theta ) {\textsf{D}}_{\theta } f$, where *G* is the matrix of coefficients of the metric. In (9), the mirror map ${\textsf{D}} \phi$ eliminates the need to compute $G_0^{-1}$ because $G_0 = {\textsf{D}}^2 \phi$ is the Jacobian of the mirror map. Here, we show that a similar property holds for the conformal mirror descent under the $\lambda$-mirror map. We let $I_d$ denote the $d \times d$ identity matrix.

#### Theorem 1

(Dynamics under the $\lambda$-mirror map) Consider the flow (21). Let $\eta _t = {\textsf{D}}^{(c_{\lambda })}_{\theta }\varphi (\theta _t)$ be the dual variable under the $\lambda$-mirror map (10). Then

22 $$\begin{aligned} \begin{aligned} {\frac{\text {d}}{\text {d}{t}}}\eta _t = -\Pi _{\lambda }(\theta _t) (I_d + \lambda \eta _t \theta _t^{\top }) {\textsf{D}}_{\theta } f(\theta _t), \end{aligned} \end{aligned}$$

where $$\Pi _{\lambda }(\theta ) := 1 + \lambda {\left\langle \theta , {\textsf{D}}^{(c_{\lambda })}_{\theta }\varphi (\theta )\right\rangle } = 1 + \lambda {\left\langle \theta , \eta \right\rangle }$$.

#### Proof

Under the primal coordinate system, we have

$$\begin{aligned} \begin{aligned} (G_{\lambda }(\theta ))_{ij}&= - \left. \frac{\partial ^2}{\partial \theta ^i \partial \theta ^{'j}}{} \textbf{L}_{\lambda , \varphi }[\theta : \theta '] \right| _{\theta = \theta '} \\&= - \left. \frac{\partial ^2}{\partial \theta ^i \partial \theta ^{'j}} \left\{ \varphi (\theta ) + \psi (\eta ') - \frac{1}{\lambda } \log (1 + \lambda {\left\langle \theta , \eta '\right\rangle }) \right\} \right| _{\theta = \theta '}\\&= \left. \frac{\partial ^2}{\partial \theta ^i \partial \theta ^{'j}} \left\{ \frac{1}{\lambda } \log (1 + \lambda {\left\langle \theta , \eta '\right\rangle }) \right\} \right| _{\theta = \theta '}\\&= \frac{1}{\Pi _{\lambda }(\theta )} \left\{ \frac{\partial \eta ^i}{\partial \theta ^j} - \frac{\lambda }{\Pi _{\lambda }(\theta )} \eta ^i \sum _{k = 1}^d \theta ^k \frac{\partial \eta ^k}{\partial \theta ^j} \right\} , \end{aligned} \end{aligned}$$

where the first equality holds by construction (see e.g., [2, Section 6.2]) and the second equality follows from the self-dual representation (17). In matrix form, we have

23 $$\begin{aligned} G_{\lambda }(\theta ) = \frac{1}{\Pi _{\lambda }(\theta )} \left( I_d - \frac{\lambda }{\Pi _{\lambda }(\theta )} \eta \theta ^{\top } \right) \frac{\partial \eta }{\partial \theta }(\theta ), \end{aligned}$$

where $\left( \frac{\partial \eta }{\partial \theta }\right) _{ij} = \frac{\partial \eta ^i}{\partial \theta ^j}$ is the Jacobian of the transformation $\theta \mapsto \eta$. Now we may invert (23) using the Sherman-Morrison formula to get

$$\begin{aligned} G_{\lambda }^{-1}(\theta ) = \Pi _{\lambda }(\theta ) \frac{\partial \theta }{\partial \eta }(\eta ) (I_d + \lambda \eta \theta ^{\top }). \end{aligned}$$

By definition, the gradient flow (21) is given by

$$\begin{aligned} {\frac{\text {d}}{\text {d}{t}}} \theta _t = -G_{\lambda }^{-1}(\theta _t) {\textsf{D}}_{\theta } f(\theta _t). \end{aligned}$$

Expressing the flow in terms of the dual variable, we have, by the chain rule again,

$$\begin{aligned} \begin{aligned} {\frac{\text {d}}{\text {d}{t}}} \eta _t&= \frac{\partial \eta }{\partial \theta }(\theta _t) {\frac{\text {d}}{\text {d}{t}}} \theta _t \\&= - \frac{\partial \eta }{\partial \theta }(\theta _t) \Pi _{\lambda }(\theta _t) \frac{\partial \theta }{\partial \eta }(\eta _t) (I_d + \lambda \eta _t \theta _t^{\top }) {\textsf{D}}_{\theta } f(\theta _t) \\&= - \Pi _{\lambda }(\theta _t) (I_d + \lambda \theta _t \eta _t^{\top }) {\textsf{D}}_{\theta } f(\theta _t). \end{aligned} \end{aligned}$$

$$\square $$

In other to implement (22), we require that the $\lambda$-mirror map and its inverse can be computed. Next, by using the fact that *g* is a conformal Hessian metric, we show that the conformal mirror descent gradient flow can be viewed as a time change of a Hessian gradient flow.

#### Theorem 2

(Time-change of Hessian gradient flow) Let $({\tilde{\theta }}_s)_{s \ge 0}$ be the Hessian gradient flow (9) with respect to the Bregman generator $\Phi _{\lambda } = \frac{1}{\lambda }(e^{\lambda \varphi } - 1)$. Consider the time change $s = s_t$, where ${\frac{\text {d}}{\text {d}{t}}} s_t = \exp (\lambda \varphi ({\tilde{\theta }}_{s_t}))$. Then $\theta _t = {\tilde{\theta }}_{s_t}$ is the conformal mirror descent (21) induced by $\varphi$. In particular, let $\zeta _t = {\textsf{D}} \Phi _{\lambda }(\theta _t)$ be the dual variable with respect to the Bregman generator $\Phi _{\lambda }$. Then the flow can be expressed as ${\frac{\text {d}}{\text {d}{t}}} \zeta _t = - e^{\lambda \varphi (\theta _t)} {\textsf{D}}_{\theta } f(\theta _t)$.

#### Proof

By (21) and (16), we have

24 $$\begin{aligned} {\frac{\text {d}}{\text {d}{t}}} \theta _t = - G_{\lambda }^{-1}(\theta _t) {\textsf{D}}_{\theta } f(\theta _t) = - e^{\lambda \varphi (\theta _t)} {\tilde{G}}_0^{-1}(\theta _t) {\textsf{D}}_{\theta } f(\theta _t), \end{aligned}$$

where ${\tilde{G}}_0 = {\textsf{D}}_{\theta }^2 \Phi _{\lambda }$. Let ${\tilde{\theta }}(s)$ be the Hessian gradient flow (9) induced by the metric ${\tilde{G}}_0$, and let $s = s_t$ be the given time change. Applying the chain rule in (9), we have

$$\begin{aligned} \begin{aligned} {\frac{\text {d}}{\text {d}{t}}} {\tilde{\theta }}_{s_t}&= {\frac{\text {d}}{\text {d}{s}}} {\tilde{\theta }}_{s_t} {\frac{\text {d}}{\text {d}{t}}} s_t = - {\tilde{G}}_0^{-1}({\tilde{\theta }}_{s_t}) {\textsf{D}}_{\theta } f({\tilde{\theta }}_{s_t}) {\frac{\text {d}}{\text {d}{t}}} s_t \\&= - e^{\lambda \varphi ({\tilde{\theta }}_{s_t})} {\tilde{G}}_0^{-1}({\tilde{\theta }}_{s_t}) {\textsf{D}}_{\theta } f({\tilde{\theta }}_{s_t}). \end{aligned} \end{aligned}$$

Comparing this with (24), we see that ${\tilde{\theta }}_{s_t} = \theta _t$. The proof of the last statement is similar. $\square$

By Theorem 2, the trajectory of a conformal mirror descent gradient flow is the same as that of a Hessian gradient flow: the conformal transformation of the metric introduces a *time-varying learning rate* depending on the value $\varphi (\theta _t)$. To implement conformal mirror descent in practice, the flow (21) must be discretized. From Definition 3 and Theorems 1 and 2, we have the following three forward Euler discretizations:

- Primal Euler discretization: $\theta _{k+1} = \theta _k - \delta G_{\lambda }^{-1}(\theta _k) {\textsf{D}}_{\theta } f(\theta _k)$.
- Dual Euler discretization: $\eta _{k+1} = \eta _k - \delta \Pi _\lambda (\theta _k)\left( I_d + \lambda \theta _{k}\eta _k^{\top } \right) {\textsf{D}}_{\theta } f(\theta _k)$.
- Mirror descent with adaptive learning rate: $\zeta _{k+1} = \zeta _k - \delta e^{\lambda \varphi (\theta _k)} {\textsf{D}}_{\theta } f(\theta _k)$, where $\zeta _k = {\textsf{D}}_{\theta } \Phi _{\lambda }(\theta _k)$.

Even if the $\lambda$-mirror map ${\textsf{D}}^{(c_{\lambda })} \varphi$ and its inverse are available in closed form, the mirror map ${\textsf{D}} \Phi _{\lambda }$ (and its inverse) may be intractable (and vice versa). Thus, the two points of view ($\lambda$-mirror vs time change) can be quite different in implementation. In particular, our generalization offers a principled alternative to implement identical gradient flows in practice when the Bregman mirror map (and its inverse) might not be computationally tractable. Also, the conformal mirror descent dynamics and the $\lambda$-duality suggest novel choices of the generator $\varphi$ and dual coordinates that are more natural in certain problems. For example, in Sect. 4 we apply it to online natural gradient learning for some generalized exponential families. A detailed analysis of the above (and possibly other) discretization schemes is left for future research.

To close this section we give a concrete example of conformal mirror descent which generalizes [16, Theorem 5.5]. For a given regular $c_{\lambda }$-convex generator $\varphi$, consider minimizing either $f(\theta ) = \textbf{L}_{\lambda , \varphi }[\theta ^* : \theta ]$ or $f(\theta ) = \textbf{L}_{\lambda , \varphi }[\theta : \theta ^*]$ for some $\theta ^* \in \Theta$. Note that *f* is typically not convex in $\theta$ (or $\eta$). We show that the conformal mirror descent evolves along geodesics of the underlying dualistic structure. See [33] for a detailed analysis of the dually flat case.

#### Proposition 3

(Primal and dual flows)

(i) The trajectory of the primal flow
  25 $$\begin{aligned} {\frac{\text {d}}{\text {d}{t}}} \theta _t = -\textrm{grad}_{G_{\lambda }} \textbf{L}_{\lambda , \varphi }[\theta ^*, \cdot ](\theta _t) \end{aligned}$$
  follows a time-changed primal geodesic, i.e., along the straight line from $\theta _0$ to $\theta ^*$ under the primal coordinate system.
(ii) The trajectory of the dual flow
  26 $$\begin{aligned} {\frac{\text {d}}{\text {d}{t}}} \theta _t = -\textrm{grad}_{G_{\lambda }} \textbf{L}_{\lambda , \varphi }[\cdot , \theta ^*](\theta _t) \end{aligned}$$
  follows a time-changed dual geodesic, i.e., along the straight line from $\eta _0$ to $\eta ^*$ under the dual coordinate system.

#### Proof

We first consider the dual flow (26). Using the self-dual representation (15),

$$\begin{aligned} {\textsf{D}}_{\theta } \textbf{L}_{\lambda , \varphi }[\cdot : \theta ^*] = {\textsf{D}}_{\theta } \varphi (\theta ) - \frac{\eta ^*}{1 + \lambda {\left\langle \theta , \eta ^*\right\rangle }} = \frac{\eta }{1 + \lambda {\left\langle \theta , \eta \right\rangle }} - \frac{\eta ^*}{1 + \lambda {\left\langle \theta , \eta ^*\right\rangle }}, \end{aligned}$$

where the last equality can be verified using the definition (10) of the $\lambda$-mirror map.

By Theorem 1 we have, after some simplification,

27 $$\begin{aligned} {\frac{\text {d}}{\text {d}{t}}} \eta _t = - \frac{1 + \lambda {\left\langle \theta _t, \eta _t\right\rangle }}{1 + \lambda {\left\langle \theta _t, \eta ^*\right\rangle }} (\eta _t - \eta ^*). \end{aligned}$$

Thus, the dual flow evolves along a time-changed dual geodesic.

Since $\textbf{L}_{\lambda , \varphi }[\theta ^* : \theta ] = \textbf{L}_{\lambda , \psi }[\eta : \eta ^*]$ and both $\textbf{L}_{\lambda , \varphi }$ and $\textbf{L}_{\lambda , \psi }$ induce the same Riemannian metric, the primal flow (25) for $\textbf{L}_{\lambda , \varphi }$ is equivalent to the dual flow for $\textbf{L}_{\lambda , \psi }$. By the case proved above, we have that the trajectory follows a time-changed straight line under the $\theta$-coordinates. $\square$

### Convergence results

In this subsection, we present continuous time convergence results for conformal mirror descent that are analogous to those of mirror descent. Our main tool is Lyapunov analysis following [34]. In what follows, we let $(\theta _t)_{t \ge 0}$ be the solution to the gradient flow (21) for a given continuously differentiable and convex function $f: \Theta \rightarrow \mathbbm {R}$. We also let $\theta ^*$ be a minimizer of *f* over $\Theta$.

We first observe that the $\lambda$-logarithmic divergence is a Lyapunov function of the gradient flow.

#### Lemma 3.1

The functional ${\mathcal {E}}_t = \textbf{L}_{\lambda , \varphi }[\theta ^* : \theta _t]$ is a Lyapunov function of the gradient flow, i.e., ${\frac{\text {d}}{\text {d}{t}}} {\mathcal {E}}_t \le 0$.

#### Proof

Using the self-dual representation (15), we have

$$\begin{aligned} \begin{aligned} {\frac{\text {d}}{\text {d}{t}}} {\mathcal {E}}_t&= {\frac{\text {d}}{\text {d}{t}}} \left( \varphi (\theta ^*) + \psi (\eta _t) - \frac{1}{\lambda } \log (1 + \lambda \langle \theta ^*, \eta _t \rangle ) \right) \\ {}&= \frac{{\left\langle \theta _t, {\frac{\text {d}}{\text {d}{t}}} \eta _t\right\rangle }}{1 + \lambda {\left\langle \theta _t, \eta _t\right\rangle }} - \frac{{\left\langle \theta ^*, {\frac{\text {d}}{\text {d}{t}}} \eta _t\right\rangle }}{1 + \lambda {\left\langle \theta ^*, \eta _t\right\rangle }}. \end{aligned} \end{aligned}$$

Using (22) and simplifying, we have

28 $$\begin{aligned} {\frac{\text {d}}{\text {d}{t}}} {\mathcal {E}}_t = \frac{1 + \lambda {\left\langle \theta _t, \eta _t\right\rangle }}{1 + \lambda {\left\langle \theta ^*, \eta _t\right\rangle }} {\left\langle {\textsf{D}}_{\theta } f(\theta _t), \theta ^* - \theta _t\right\rangle } \le \frac{1 + \lambda {\left\langle \theta _t, \eta _t\right\rangle }}{1 + \lambda {\left\langle \theta ^*, \eta _t\right\rangle }} (f(\theta _t) - f(\theta ^*)) \le 0.\nonumber \\ \end{aligned}$$

$$\square $$

#### Theorem 4

Define $\tau _t = \int _0^t e^{\lambda \varphi (\theta _s)} \text {d}{s}$, so that ${\dot{\tau }}_t = {\frac{\text {d}}{\text {d}{t}}} \tau _t = e^{\lambda \varphi (\theta _t)}$. Let ${\hat{\theta }}_t = \frac{1}{\tau _t} \int _0^t \theta _s {\dot{\tau }}_s \text {d}{s}$, which is a weighted average of the trajectory up to time *t*. If $\theta ^*$ is a minimizer of *f* over $\Theta$, then

29 $$\begin{aligned} f({\hat{\theta }}_t) - f(\theta ^*) \le \frac{\textbf{B}_{\Phi _{\lambda }}[\theta ^* : \theta _0]}{\tau _t}, \end{aligned}$$

where $\Phi _{\lambda } = \frac{1}{\lambda } (e^{\lambda \varphi } - 1)$ is the Bregman generator. In particular, if *f* is strictly convex, then $f({\hat{\theta }}_t) - f(\theta ^*) = O(\frac{1}{t})$ as $t \rightarrow \infty$.

#### Proof

This result can be derived using Theorem 2 and convergence results of Hessian gradient flow (see e.g. [17, Section 2.1.3]). For completeness, we give a self-contained proof. Using a similar argument as in the proof of Lemma 3.1, we have that

$$\begin{aligned} {\mathcal {E}}_t = \frac{1}{\lambda } \left( 1 - e^{-\lambda \textbf{L}_{\lambda , \varphi }[\theta ^* : \theta _t]}\right) + \int _0^t e^{\lambda (\varphi (\theta _s) - \varphi (\theta ^*))} (f(\theta _s) - f(\theta ^*)) \text {d}{s} \end{aligned}$$

satisfies

30 $$\begin{aligned} {\frac{\text {d}}{\text {d}{t}}} {\mathcal {E}}_t = - e^{-\lambda (\varphi (\theta _t) - \varphi (\theta ^*))} \textbf{B}_{\Phi _{\lambda }}[\theta ^* : \theta _t] \le 0, \end{aligned}$$

and hence is another Lyapunov function. Since ${\mathcal {E}}_t$ is non-increasing, we have

31 $$\begin{aligned} e^{-\varphi (\theta ^*)} \tau _t \int _0^t \frac{{\dot{\tau }}_s}{\tau _t} (f(\theta _s) - f(\theta ^*)) \text {d}{s} \le {\mathcal {E}}_t \le {\mathcal {E}}_0 = \frac{1}{\lambda } (1 - e^{-\lambda \textbf{L}_{\lambda , \varphi }[\theta ^* : \theta _0]}). \end{aligned}$$

Note that by (19), the last expression in (31) is equal to $e^{-\varphi (\theta ^*)} \textbf{B}_{\Phi _{\lambda }}[\theta ^* : \theta _0]$. Since $f(\cdot ) - f(\theta ^*)$ is convex, by Jensen’s inequality we have

$$\begin{aligned} f({\hat{\theta }}_t) - f(\theta ^*) \le \int _0^t \frac{{\dot{\tau }}_s}{\tau _t} (f(\theta _s) - f(\theta ^*)) \text {d}{s} \le \frac{1}{\tau _t}{} \textbf{B}_{\Phi _{\lambda }}[\theta ^* : \theta _0]. \end{aligned}$$

If *f* is strictly convex, from (28) we have that $\lim _{t \rightarrow \infty } \theta _t = \theta ^*$. Since $e^{\lambda \varphi (\theta _t)} \rightarrow e^{\lambda \varphi (\theta ^*)}$, the quantity $\tau _t = \int _0^t e^{\lambda \varphi (\theta _s)} \text {d}{s}$ grows linearly as $t \rightarrow \infty$. It follows from (29) that $f({\hat{\theta }}_t) - f(\theta ^*) = O(\frac{1}{t})$ as $t \rightarrow \infty$. $\square$

## Online estimation of generalized exponential family

Mirror descent is often used to estimate parameters of stochastic models, both offline and online. Using a duality between the exponential family and Bregman divergence [6], the authors of [14] considered online parameter estimation for exponential families, and showed that the mirror descent step is equivalent to the natural gradient step [35]. In this section, we generalize this result to obtain tractable online learning algorithms for the $\lambda$-*exponential family* introduced in [19]. In particular, it includes heavy-tailed distributions, such as the *t*-distribution, which cannot be expressed as exponential families.

We begin with some preliminaries. Following [19], by a $\lambda$-*exponential family* we mean a parameterized probability density (with respect to a reference measure $\nu$) of the form

32 $$\begin{aligned} p_{\theta }(x) = (1 + \lambda \langle \theta , F(x) \rangle )_+^{1/\lambda } e^{-\varphi (\theta )}, \end{aligned}$$

where $x_+ = \max \{x, 0\}$ and $F(x) = (F^1(x), \ldots , F^d(x))$ is a vector of statistics. For example, if $\nu$ is the Lebesgue measure on $\mathbbm {R}$, $\lambda \in (-2, 0)$ and $F(x) = (x, x^2)$, then we obtain from (32) the Student’s *t* distribution (as a location-scale family) with $\frac{-2}{\lambda } - 1 > 0$ degrees of freedom (see Example 1 below). The density (32) is a generalized or deformed exponential family and is a reparameterized version of the *q*-*exponential family* (where $q = 1 - \lambda$) in statistical physics (see [19, Section 3] for the precise relation).^5^ As $\lambda \rightarrow 0$, we recover the usual exponential family. Under suitable regularity conditions (including the restriction $\lambda < 1$ or equivalently $q = 1 - \lambda > 0$), it can be shown that the divisive normalization function $\varphi$ in (32) is $c_{\lambda }$-convex on the parameter space $\Theta$ and hence defines a $\lambda$-logarithmic divergence. This divergence can be interpreted probabilistically as $\textbf{L}_{\lambda , \varphi }[ \theta : \theta '] = {\textsf{H}}^{\textsc {r}}_q ( p_{\theta '} \vert \vert p_{\theta })$, where ${\textsf{H}}^{\textsc {r}}_q$ is the *Rényi divergence* of order *q*. Consequently, the induced Riemannian metric is a constant multiple of the *Fisher information metric* ${\mathcal {I}}$ [37]:

33 $$\begin{aligned} G_{\lambda }(\theta ) = (1 - \lambda ) {\mathcal {I}}(\theta ). \end{aligned}$$

Moreover, the dual variable $\eta = {\textsf{D}}_{\theta }^{(c_{\lambda })} \varphi (\theta )$ under the $\lambda$-mirror map can be interpreted as a generalized expectation parameter known as the *escort expectation*:

$$\begin{aligned} \eta = \int F(x) \frac{p_{\theta }(x)^q}{ \int p_{\theta }^{q} \text {d}{\nu }} \text {d}{\nu }(x). \end{aligned}$$

In fact, the density (32) maximizes the Rényi entropy of order *q* subject to constraints on the escort expectation. These (and other) results nicely parallel those of the exponential family (see e.g. [2, Chapter 2]).

We now consider the online estimation of (32) under i.i.d. sampling. By considering the distribution of $Y = F(X)$, we have a $\lambda$-exponential family on (a subset of) ${\mathbb {R}}^d$ of the form

34 $$\begin{aligned} p_{\theta }(y) = (1 + \lambda {\left\langle \theta , y\right\rangle })_+^{1/\lambda } e^{-\varphi (\theta )}. \end{aligned}$$

Suppose we observe data points $y_k$, $k = 1, 2, \ldots$. Let the current guess of the parameter be $\theta _k$. After observing $y_k$, we update $\theta _k$ to $\theta _{k+1}$ by a minimizing gradient step with respect to the log-loss

35 $$\begin{aligned} f_k(\theta ) = - \log p_{\theta }(y_k) = \varphi (\theta ) - \frac{1}{\lambda } \log (1 + \lambda {\left\langle \theta , y_k\right\rangle }). \end{aligned}$$

Note that the negative log-likelihood $f_k$ is typically not convex in $\theta$. We do this by discretizing the conformal mirror descent (22), where the generating function $\varphi$ is the potential function in (34). Since $G_{\lambda }$ is a multiple of the Fisher metric, the forward Euler step of (22) in dual coordinates leads to the (unconstrained) *natural gradient update*

36 $$\begin{aligned} \eta _{k+1} = \eta _k - \delta _k \Pi _{\lambda }(\theta _k) (I_d + \lambda \eta _k \theta _k^{\top }) {\textsf{D}}_{\theta } f_k(\theta _k), \end{aligned}$$

where $\delta _k > 0$ is the learning rate. Simplifying (36), we obtain an explicit and clean update that is not obvious from the time change perspective.

### Theorem 5

(Online natural gradient step for $\lambda$-exponential family) The online natural gradient update (36) is given by

37 $$\begin{aligned} \eta _{k+1} = \eta _k + \delta \frac{1 + \lambda {\left\langle \theta _k, \eta _k\right\rangle }}{1 + \lambda {\left\langle \theta _k, y_k\right\rangle } } (y_k - \eta _k). \end{aligned}$$

### Proof

Differentiating $f_k(\theta )$ in (35), we have

$$\begin{aligned} {\textsf{D}}_{\theta }f_k(\theta ) = \frac{\eta }{1 + \lambda {\left\langle \theta , \eta \right\rangle }} - \frac{y_k}{1 + \lambda {\left\langle \theta , y_k\right\rangle }}, \end{aligned}$$

which has the same form as in the dual gradient flow in Proposition 3(ii). (This is not a coincidence in view of the duality between $\lambda$-exponential family and $\lambda$-logarithmic divergence; see [19, Section VI].) Continuing the computation as in the proof of Proposition 3, we obtain (37) which is the discrete analogue of (27). $\square$

Since (36) is a natural gradient update, by [35, Theorem 2] the algorithm (when $\delta _k = \frac{1}{k}$) is Fisher efficient as $k \rightarrow \infty$. When $\lambda \rightarrow 0$, we recover the linear update for exponential families derived in [14]. In general, an extra projection step, which is also necessary for the exponential family ($\lambda = 0$), is needed to constrain $\theta _{k+1} \in \Theta$ (or $\eta _{k+1} \in H$). We use clipping and reflection across the boundary to enforce the domain constraints in our experiments below.

**Fig. 2:**
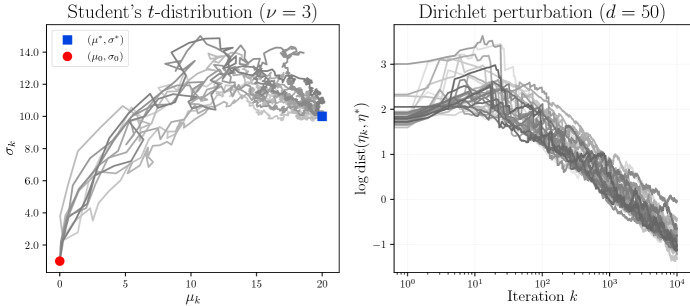
Left: 10 trajectories of (37) for the Student *t*-distribution location scale family (38) with $\nu = 3$ degrees of freedom, where the learning rate is $\delta _k = 1/k$. We show the dynamics of $(\mu _k, \sigma _k)$ over a sample of 10000 data points. Red dot: initial guess $(\mu _0, \sigma _0)$. Blue square: true parameter $(\mu ^*, \sigma ^*)$. Right: Plot of $\log \textrm{dist}(\eta _{k}, \eta ^{*})$ against $\log _{10} k$ for the Dirichlet perturbation model (Example 2) over 30 trajectories of (37), each with 10000 data points. Here $\textrm{dist}(\eta , \eta ') = \vert \log (\eta ) - \log (\eta ')\vert$ is used as a metric on the dual domain $H = (0, \infty )^d$. In this simulation, $d = 50$, $\lambda = -0.3$ and $\delta _k = 1/k$. We observe that $\textrm{dist}(\eta _k, \eta ^{*})$ decays like $O(k^{-1/2})$, which is consistent with the asymptotic efficiency of online natural gradient learning

### Example 1

(*Student’s* *t*-*distribution as a location-scale family*) For a fixed $\nu > 0$, the Student’s *t*-distribution with $\nu$ degrees of freedom has density on $\mathbbm {R}$ given by

38 $$\begin{aligned} p(x ;\mu ,\sigma , \nu ) = \frac{\Gamma ((\nu + 1)/2)}{\Gamma (\nu /2) \sqrt{\nu \pi } \sigma }\left( 1 + \frac{1}{\nu } \frac{(x-\mu )^2}{\sigma ^2} \right) ^{-(\nu +1)/2}, \end{aligned}$$

where $\mu$ and $\sigma$ are the location and scale parameters, respectively, and $\Gamma$ is the gamma function.^6^ In the following, we regard $\nu$ as known and consider online estimation of $(\mu , \sigma )$.

Let $\lambda = \tfrac{-2}{\nu + 1} \in (-2, 0)$ and $F(x) = (x, x^2)^{\top }$. Then we can express (38) as a $\lambda$-exponential family $p_{\theta }(x) = (1 + \lambda {\left\langle \theta , F(x)\right\rangle })^{1/\lambda } e^{-\varphi (\theta )}$. The natural parameter $\theta$ is given by

$$\begin{aligned} \theta = (\theta ^1, \theta ^2) = \left( \dfrac{2 \mu }{- \lambda \mu ^{2} + \sigma ^{2} \left( \lambda + 2\right) }, -\dfrac{1}{- \lambda \mu ^{2} + \sigma ^{2} \left( \lambda + 2\right) }\right) , \end{aligned}$$

and takes values in the set

$$\begin{aligned} \Theta = \left\{ \theta = (\theta ^1,\theta ^2) \in \mathbbm {R}^2 : \theta ^2 < 0 \text { and } \lambda (\theta ^1)^2 - 4\theta ^2 > 0\right\} . \end{aligned}$$

The potential function $\varphi$ is given on $\Theta$ by

$$\begin{aligned} \varphi (\theta ) = \log {\left( \frac{\sqrt{\frac{\lambda (\theta ^1)^{2} - 4 \theta ^{2}}{\lambda + 2}}}{-2 \theta ^{2}} \right) } - \frac{1}{\lambda }\log {\left( \frac{-4 \theta ^{2}}{\lambda (\theta ^1)^{2} - 4 \theta ^{2}} \right) } + C, \end{aligned}$$

where *C* is a constant depending only on $\nu$. By a straightforward computation, we obtain explicit expressions of the $\lambda$-mirror map and its inverse:

$$\begin{aligned} \eta&= {\textsf{D}}_{\theta }^{(c_{\lambda })}\varphi _{\lambda }(\theta ) = \left( \frac{-\theta ^{1}}{2 \theta ^{2}}, \frac{\lambda (\theta ^{1})^{2} + (\theta ^1)^{2} - 2 \theta ^{2}}{2 (\lambda + 2) \theta _{2}^{2} } \right) ,\\ \theta&= {\textsf{D}}_{\eta }^{(c_{\lambda })}\psi (\eta ) = \left( \frac{-2 \eta ^{1}}{2 (\lambda + 1) (\eta ^1)^2 - (\lambda + 2) \eta ^{2}} , \frac{1}{2 (\lambda + 1) (\eta ^1)^2 - (\lambda + 2) \eta ^{2}}\right) . \end{aligned}$$

In Figure (left), we show ten trajectories (in terms of $(\mu _k, \sigma _k)$) of the algorithm (37) with $\delta _k = 1/k$, where the true parameter is $(\mu ^*, \sigma ^*)$ and the initial guess is $(\mu _0, \sigma _0)$. As expected, the iterates converge to $(\mu ^*, \sigma ^*)$ as $k \rightarrow \infty$. The preceding computations can be generalized to the multivariate location-scale *t*-distribution where the degrees of freedom is also assumed to be known.

### Example 2

(*Dirichlet perturbation on the unit simplex*) The Dirichlet perturbation model is a fundamental example of the $\lambda$-exponential family (see [19, Example 3.14]) and is closely related to the *Dirichlet optimal transport problem* studied in [15, 16, 26]; see also Sect. 5 below, where we use the Dirichlet transport to define gradient flows on the simplex. This model, which is also called the *shifted Dirichlet distribution*, has been applied in compositional data analysis (see [38]).

Fix $d \ge 1$ and consider the open unit simplex

39 $$\begin{aligned} \triangle ^{1+d} = \left\{ p = (p^0, p^1, \ldots , p^d) \in (0, 1)^{1+d}: \sum _{i = 0}^d p^i = 1 \right\} . \end{aligned}$$

Given $p, q \in \triangle ^{1 + d}$, define the *perturbation operation* by

40 $$\begin{aligned} p \oplus q = \left( \frac{p^0 q^0}{\sum _{j=0}^d p^j q^j}, \ldots , \frac{p^d q^d}{\sum _{j=0}^d p^j q^j} \right) . \end{aligned}$$

This is the vector addition operation under the *Aitchison geometry* in compositional data analysis [39]. Let $\sigma > 0$ and let $\lambda = -\sigma < 0$. Fix $p \in \triangle ^{1 + d}$, which we regard as the unknown parameter, and let $D = (D^0, \ldots , D^d)$ be a random vector whose distribution is the Dirichlet distribution with parameters $(\sigma ^{-1}/(1 + d), \ldots , \sigma ^{-1}/(1 + d)) \in (0, \infty )^{1+d}$. As $\sigma \rightarrow 0$, the distribution of *D* converges weakly to the point mass at the barycenter $(1/(1 + d), \ldots , 1/(1 + d))$. Thus, we may regard $\sigma$ as a noise parameter. The Dirichlet perturbation model is specified as

41 $$\begin{aligned} Q = p \oplus D. \end{aligned}$$

It may be regarded as a multiplicative analogue of the Gaussian additive model $Y = X + \epsilon$, where $\epsilon \sim N(0, \sigma ^2 I_d)$. Alternatively, we may think of (41) as a natural additive (but non-Gaussian) noise model under the Aitchison geometry.

By [19, Proposition 3.16], the distribution of *Q* can be expressed as a $\lambda$-exponential family with $\lambda = -\sigma < 0$, if we let $F(q) = (q^1/q^0, \ldots , q^d/q^0)$ and $\theta = (p^0/\lambda p^1, \ldots , p^0/\lambda p^d) \in \Theta = (-\infty , 0)^d$. By [19, (III.30)], the potential function is given by

$$\begin{aligned} \varphi (\theta ) = \frac{1}{\lambda (1 + d)} \sum _{i = 1}^d \log (-\theta ^i). \end{aligned}$$

Letting $d = 1$ and $\lambda = -1$ (and replacing $\theta$ by $-\theta$), recovers the first example in Table 1. The dual variable $\eta$ is given by $\eta ^i = \frac{1}{\lambda \theta ^i} = \frac{p^i}{p^0}$. In Figure 2 (right), we illustrate the $O(k^{-1/2})$ convergence rate of the online estimation algorithm (37). In fact, it can be verified that the update (37), when expressed in terms of $p_k$ (the current estimate of *p*) and $q_{k}$ (the new data point) with values in $\triangle ^{1 + d}$, is independent of the value of $\lambda < 0$. Thus, for online estimation of the Dirichlet perturbation model, we may treat $\sigma > 0$ (or $\lambda < 0$) as *unknown*.

## Gradient flows on the simplex via Dirichlet transport

By Brenier’s theorem [40], the mirror map $\zeta = {\textsf{D}} \phi (\theta )$ in classical (Bregman) mirror descent can be interpreted as an optimal transport map for the quadratic cost $c(x, y) = \frac{1}{2}|x - y|^2$. Also, the Bregman divergence is the *c*-divergence of the quadratic cost. This suggests an interpretation of mirror descent in terms of optimal transport. Our conformal mirror descent generalizes this set-up to the logarithmic cost $c_{\lambda }(x, y) = \frac{-1}{\lambda } \log (1 + \lambda {\left\langle x, y\right\rangle })$ for $\lambda \ne 0$. In this section, we specialize to the unit simplex and the case $\lambda = -1$. Using the *Dirichlet optimal transport problem* studied in [26], we define a family of gradient flows on the unit simplex and compare them with the entropic descent, which is an important and practical example of mirror descent.

### Dirichlet transport

Following [26], we define the *Dirichlet cost function* on $\triangle ^{n} \times \triangle ^{n}$ (where $n = 1 + d \ge 2$) by

42 $$\begin{aligned} c(p, q) = \log \left( \sum _{i = 0}^{n-1} \frac{1}{n} \frac{q^i}{p^i} \right) - \sum _{i = 0}^{n-1} \frac{1}{n} \log \frac{q^i}{p^i}. \end{aligned}$$

It is closely related to the Dirichlet perturbation model in Example 2, because the density of *Q* (with respect to a suitable reference measure) is proportional to $e^{c(p, q)/\lambda }$ [26, Remark 6]. It is easy to verify that $c(p, q) = \textbf{L}_{-1, \varphi }[q : p]$, where $\varphi (p) = - \sum _{i = 0}^{n-1} \frac{1}{n} \log p^i$ is $c_{-1}$-convex on $\triangle ^n$. Up to a change of variables and addition of linear terms (see [17, Remark 3]), the Dirichlet cost function is equivalent to the logarithmic cost $c_{-1}$. The ($-1$)-mirror map then corresponds to the optimal transport map of the Dirichlet transport. We now adapt the logarithmic divergence and the $(-1)$-mirror map to the simplex following the notations of [26]. The role of the $c_{-1}$-convex generator is now played by an *exponentially concave* function.

#### Definition 4

(*Exponentially concave function*) A smooth function $\varphi : \triangle ^n \rightarrow \mathbbm {R}$ is said to be exponentially concave if $e^{\varphi }$ is concave. Given such a function, we define its *L*-divergence by

43 $$\begin{aligned} \textbf{L}_{\varphi }[q : p] = \log ( 1 + {\left\langle {\textsf{D}} \varphi (p), q - p\right\rangle }) - (\varphi (q) - \varphi (p)). \end{aligned}$$

It is easy to see that if $\varphi$ is exponentially concave, then $-\varphi$ is $c_{-1}$-convex and $\textbf{L}_{\varphi } = \textbf{L}_{-1, -\varphi }$. In order that the induced Riemannian metric is well-defined, we assume that ${\textsf{D}}^2 e^{\varphi }$ is strictly negative definite when restricted to the tangent space of $\triangle ^{n}$. The ($-1$)-mirror map is now given in terms of the optimal transport map of the Dirichlet transport. Directional derivatives of a differentiable function *f* on $\triangle ^n$ are defined by

$$\begin{aligned} \widetilde{{\textsf{D}}}_i f(p) = {\left\langle {\textsf{D}} f(p), e_i-p\right\rangle }, \quad 0 \le i \le n - 1, \end{aligned}$$

where $\left\{ e_i\right\} _{i=0}^{n-1}$ denotes the standard Euclidean basis. In conjunction with the perturbation operator (40), the *powering operator* on $\triangle ^n$ is defined as

$$\begin{aligned} \alpha \otimes p = \left( \frac{(p^0)^{\alpha }}{\sum _{j = 0}^{n-1} (p^j)^{\alpha }}, \ldots , \frac{(p^{n-1})^{\alpha }}{\sum _{j = 0}^{n-1} (p^j)^{\alpha }} \right) , \quad p \in \triangle ^n,\ \alpha \in \mathbbm {R}. \end{aligned}$$

Note that $\triangle ^n$ is an $(n - 1)$-dimensional real vector space under the operations $\oplus$ and $\otimes$. We define $\ominus p = (-1) \otimes p$ to be the additive inverse of *p*.

#### Definition 5

(*Portfolio and optimal transport maps*) Given the exponentially concave generator $\varphi$, we define the portfolio map $\pi _{\varphi } : \triangle ^n \rightarrow \triangle ^n$ by

44 $$\begin{aligned} (\pi _{\varphi }(p))^i = p^i \left( 1 + \widetilde{{\textsf{D}}}_i \varphi (p) \right) , \quad 0 \le i \le n - 1. \end{aligned}$$

The optimal transport map $T_{\varphi }: \triangle ^n \rightarrow \triangle ^n$ is defined by

45 $$\begin{aligned} q = T_{\varphi }(p) = p \oplus \pi _{\varphi }(\ominus p). \end{aligned}$$

That $T_{\varphi }$ is an optimal transport map for the Dirichlet cost function (42) is proved in [26, Theorem 1], which is an analogue of Brenier’s theorem. The terminology “portfolio map” for the mapping $\pi _{\varphi }$ is motivated by its use in portfolio theory [15, 18, 41].

#### Example 3

(*Examples of portfolio and transport maps*)

(i) Let $\varphi (p) = \sum _{i = 0}^{n-1} \frac{1}{n} \log p^i$. Then the associated portfolio map is the constant map $\pi _{\varphi }(p) = \left( \frac{1}{n}, \ldots , \frac{1}{n} \right)$ called the *equal-weighted portfolio*. From (45), the transport map is the identity $T_{\varphi }(p) = p$. This function corresponds to the self-dual quadratic function $\frac{1}{2}\vert x \vert ^2$ whose Euclidean gradient is the identity.
(ii) Let $\varphi (p) = \frac{1}{\alpha } \log \left( \sum _{j = 0}^{n-1} (p^i)^{\alpha } \right)$ where $\alpha \in (-\infty , 1)$ is a fixed parameter. Then $\pi _{\varphi }(p) = \alpha \otimes p$ is called the *diversity-weighted portfolio*. The transport map is $T_{\varphi }(p) = (1 - \alpha ) \otimes p$, and can be interpreted as a dilation under the Aitchison geometry, with $\alpha \rightarrow 0$ recovering the identity transport.

Let $f: \triangle ^n \rightarrow \mathbbm {R}$ be a differentiable function. Using the Riemannian metric *g* induced by $\textbf{L}_{\varphi }$, we can define the gradient flow

46 $$\begin{aligned} {\frac{\text {d}}{\text {d}{t}}} p_t = -\textrm{grad}_{g} f(p_t), \end{aligned}$$

which is a special case of (21) (up to reparameterization) when $\lambda = -1$. The following result is an explicit derivation of the dynamics under the dual variable $q_t = T_{\varphi }(p_t)$, defined in terms of the transport map. We omit the proof as it is a straightforward, but tedious computation.

#### Theorem 6

(Conformal mirror descent on $\triangle ^d$ under Dirichlet transport) Consider the gradient flow (46), and let $q_t = T_{\varphi }(p_t)$. For $0 \le i \le n-1$,

47 $$\begin{aligned} {\frac{\text {d}}{\text {d}{t}}} \log q_t^i = \frac{-p_t^i}{\pi _{\varphi }^i(\ominus p_t)} \left[ \widetilde{{\textsf{D}}}_i f(p_t) - q_t^i \sum _{j = 0}^{n-1} \left( \frac{p_t^j}{p_t^i}\right) ^2 \widetilde{{\textsf{D}}}_j f(p_t) \right] . \end{aligned}$$

#### Example 4

Consider the equal-weighted portfolio in (3). Then $q_t = T_{\varphi }(p_t) = p_t$, and corresponding gradient flow (47) is given by

$$\begin{aligned} {\frac{\text {d}}{\text {d}{t}}} \log \frac{p_t^i}{p_t^j} = - n \left[ p_t^i \widetilde{{\textsf{D}}}_i f(p_t) - p_t^j \widetilde{{\textsf{D}}}_j f(p_t) \right] , \quad 0 \le i,j \le n-1. \end{aligned}$$

This motivates the multiplicative discrete update:

$$\begin{aligned} p_{k+1}^i = \frac{ p_k^i \exp \left( -\delta _k p_k^i \widetilde{{\textsf{D}}}_i f(p_t)\right) }{\sum _{j = 0}^{n-1} p_k^j \exp \left( -\delta _k p_k^j \widetilde{{\textsf{D}}}_j f(p_t)\right) }. \end{aligned}$$

This is reminiscent of the *entropic descent* (Bregman mirror descent on $\triangle ^n$ induced by the negative Shannon entropy), whose update is given by

48 $$\begin{aligned} p_{k+1}^i = \frac{ p_k^i \exp \left( -\delta _k {\textsf{D}}_i f(p_t)\right) }{\sum _{j = 0}^{n-1} p_k^j \exp \left( -\delta _k {\textsf{D}}_j f(p_t)\right) }, \end{aligned}$$

where ${\textsf{D}}_i f$ is the *i*-th component of ${\textsf{D}} f$.

**Fig. 3:**
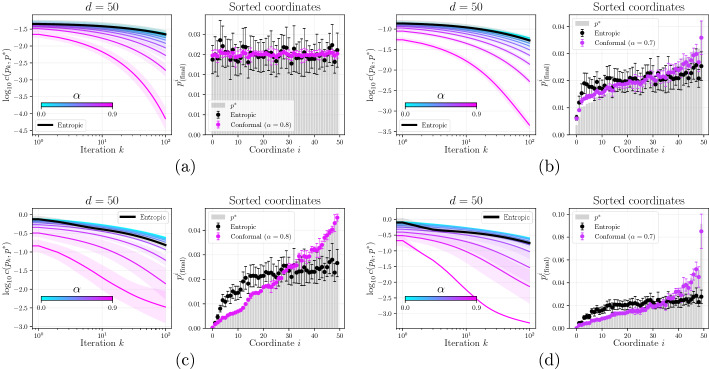
Convergence rates (left) and final estimates $p_{(\text {final})}$ (right) of $f(p_k) = c(p_k, p^*)$ for the entropic and conformal descents using step size $\delta _k = \frac{1}{d\sqrt{k}}$, for various targets $p^*$ that were randomly chosen and fixed. In Figure a, $p^*$ is the barycenter on $\triangle ^d$, whereas in Figures  3b-3d, $p^*$ was sampled from a Dirichlet distribution with varying parameters. For both the entropic and conformal descents, we plot the average over 12 randomized initial points $p_0$ (for $p^*$ fixed)

#### Example 5

Consider minimization of the function $f(p) = c(p, p^*)$ where *c* is the Dirichlet cost function defined by (42) and $p^*$ is fixed. In this experiment, we generate $p^*$ randomly according to various distributions on $\triangle ^n$. We implement (47) using the forward Euler discretization for the diversity-weighted portfolio (Example 3(ii)) where $\alpha \in \{0, \ldots , 0.9\}$, and compare the performance with that of the entropic descent (48). The results are shown in Figure . Values of $\alpha$ closer to 1 perform better than the entropic descent across all settings, and recover the minimizer $p^*$ considerably more accurately.

## Discussion and future directions

Convex duality and Bregman divergence underlie much of the theory and applications of classical information geometry. In this paper, we use the $\lambda$-duality and the associated logarithmic divergence to propose a tractable gradient flow called the conformal mirror descent. We demonstrate its usefulness in online parameter estimation and gradient flows on the simplex. Here, we discuss other related work and some directions for future research.

In this paper, we generalize the Hessian gradient flow primarily from the information-geometric point of view. Being a fundamental first-order optimization method, mirror descent has been studied and generalized in many directions. For instance, convergence of many discrete and continuous time descent algorithms was studied using Lyapunov arguments in [34]. In [42], a family of accelerated mirror descent algorithms with quadratic convergence was proposed. Likewise, [43] presents a unifying analysis of accelerated descent using variational methods. A future avenue is to explore accelerated variants of the conformal mirror flow, and to interpret these using information-geometric frameworks; one such exploration is presented by [44].

Mirror descent provides a concrete framework to understand seemingly unrelated optimization algorithms. For example, several recent works [45–47] have analyzed and interpreted the popular Sinkhorn algorithm [48]—an iterative scheme used for solving the entropic optimal transport problem [49]—as a form of mirror descent. Our conformal mirror descent may be applied to develop new algorithms for regularized optimal transport problems and analyzing their convergence properties.

Statistical inference and machine learning involving generalized exponential families is the subject of a recent line of work, for e.g. [50, 51]. We expect that $\lambda$-duality and logarithmic divergences will be useful in this endeavor. Nevertheless, the current framework (as in [19]) assumes that the curvature parameter $\lambda$ is given and known (except in special cases such as the Dirichlet perturbation model in Example 2). A natural direction is to develop data-driven methods to determine $\lambda$ (and analogous quantities for other generalized exponential families).

The $\lambda$-duality is a special case of the *c*-duality in optimal transport, where $c = c_{\lambda }$ is the logarithmic cost given by (5). While the $\lambda$-duality is particularly tractable, efficient algorithms related to general *c*-duality will likely open up many new applications. For example, the recent paper [52] used *c*-convexity to define normalizing flows on Riemannian manifolds. It is also natural to analyze similarly derived gradient flows with respect to other cost functions. We hope our results will motivate and inspire further work in applications of generalized *c*-convex duality.

## Data Availability

The data used in this paper was simulated and the codes are available upon request.

## Footnotes

1 We reserve the symbol $\eta$ for the dual variable under the $\lambda$-duality; see (10).

2 In our applications *x* and *y* only vary in respective domains such that $1 + \lambda {\left\langle x, y\right\rangle } > 0$, so the logarithm in (5) is well defined.

3 *H* is the uppercase of the Greek letter $\eta$.

4 We may let $M = \Theta$ as sets.

5 Note that parameterized densities similar to (32) were studied by other authors such as [36], but their motivations were orthogonal to ours.

6 Here the dominating measure is the Lebesgue measure on $\mathbbm {R}$ and $\nu \in (0, \infty )$ denotes the degrees of freedom.
